# What constitutes a true polymicrobial periprosthetic joint infection? From multiple detections to organism-level causality

**DOI:** 10.3389/fmicb.2026.1936444

**Published:** 2026-08-21

**Authors:** Jixin Chen, Qinxin Zhou, Yongming Zhang, Jianliang Chen, Xiaodong Zheng, Feng Ye

**Affiliations:** 1 Department of Orthopaedic Surgery, Shaoxing Shangyu District Hospital of Traditional Chinese Medicine, Shaoxing, China; 2 Department of Orthopaedic Surgery, Shaoxing Hospital of Traditional Chinese Medicine Affiliated to Zhejiang Chinese Medical University, Shaoxing, China

**Keywords:** antimicrobial stewardship, contamination, metagenomic sequencing, microbiological diagnosis, organism-level causality, periprosthetic joint infection, polymicrobial infection, sonication

## Abstract

Polymicrobial periprosthetic joint infection (PJI) is often defined by recovery of two or more microorganisms from the same clinical episode, but this numerical definition is biologically incomplete. A second organism may represent a true co-pathogen, colonization, contamination, reagent background, nonviable DNA after antimicrobial exposure, or an analytically plausible signal of uncertain clinical importance. Established PJI definitions determine whether infection is present but do not provide a validated organism-level rule for assigning causality to every detection. We therefore propose a sequential approach: first establish PJI using accepted episode-level criteria, then adjudicate each detected microorganism separately before classifying the episode as polymicrobial. This Mini Review examines evidence relevant to organism-level causal attribution, including sampling integrity, reproducibility across independent deep specimens, anatomical coherence, orthogonal confirmation, quantitative and temporal signal, organism biology, and clinical concordance. We also consider how tissue culture, synovial fluid culture, sonication, blood culture, PCR, and metagenomic sequencing generate different interpretive challenges, particularly after antimicrobial exposure. Finally, we propose a pragmatic four-category vocabulary—strongly supported participant, probable participant, uncertain detection, and likely contaminant—to make organism-level causal confidence explicit in multidisciplinary interpretation and research reporting. This framework is intended as an interpretive aid rather than a validated diagnostic score. Whether it improves inter-rater consistency, antimicrobial precision, or organism-specific outcomes requires prospective validation.

## 1 Introduction

Periprosthetic joint infection is a biofilm-associated complication in which microbiological diagnosis guides both surgery and prolonged antimicrobial therapy (Zimmerli et al., 2004; Tande and Patel, 2014; Del Pozo and Patel, 2009; Izakovicova et al., 2019). The microbiological landscape of PJI continues to evolve, with recent database and multicenter studies documenting shifting organism profiles and resistance patterns across different geographic regions (Tai et al., 2022; Peel et al., 2012; Peng et al., 2021; Hu et al., 2021; Fröschen et al., 2022). Contemporary definitions combine clinical, synovial, histological, and microbiological criteria to decide whether infection is present (Osmon et al., 2013; Parvizi et al., 2011, 2018; McNally et al., 2021). They are not designed to answer a different question that becomes critical when more than one organism is detected: which organisms are causally participating in the same infected implant ecosystem?

Polymicrobial PJI is reported in a clinically meaningful minority of episodes, and cohorts have associated it with sinus tracts, wound failure, previous procedures, enterococci, gram-negative bacilli, and lower treatment success (Marculescu and Cantey, 2008; Tan et al., 2016; Li et al., 2021; Flurin et al., 2019; Wimmer et al., 2016; Bozhkova et al., 2016; Auñón et al., 2025; Chahla et al., 2019). Yet the label is method-dependent. More specimens, sonication, prolonged incubation, polymerase chain reaction (PCR), and metagenomic sequencing all increase the number of detectable signals (Trampuz et al., 2007; Atkins et al., 1998; Peel et al., 2017; Street et al., 2017; Thoendel et al., 2018). The resulting list may include true pathogens and analytical or clinical bystanders. A robust definition therefore cannot rely solely on counting organism names.

Accordingly, this review does not propose another definition of whether PJI is present. It addresses the next interpretive step. Once infection has been established, each detected microorganism should be adjudicated separately for causal participation before the episode is classified as polymicrobial. The practical change proposed is therefore sequential. Clinicians first establish PJI using accepted episode-level criteria, then determine whether each additional detection has sufficient organism-level support to count toward a polymicrobial diagnosis. We organize established evidentiary domains around this second question and propose a four-category vocabulary to make causal confidence and uncertainty explicit in multidisciplinary interpretation and research reporting.

## 2 Literature identification and scope

This focused Mini Review was updated through targeted PubMed/MEDLINE searches from database inception through June 2026 using combinations of the terms “periprosthetic joint infection,” “prosthetic joint infection,” “polymicrobial,” “culture,” “sonication,” “PCR,” “metagenomic sequencing,” “biofilm,” and “contamination.” We prioritized guideline and consensus documents, clinical studies directly addressing polymicrobial PJI, studies evaluating specimen number and culture or sonication yield, and PJI-specific molecular diagnostic studies. General biofilm and low-biomass contamination literature was included only when it directly informed interpretation of mixed microbial detections. The review was designed as a focused narrative synthesis rather than a systematic review. No meta-analysis or formal risk-of-bias assessment was performed.

## 3 Infection status and organism causality are different questions

The first diagnostic task is to establish that the joint is infected. The second is to determine the role of each detected microorganism. These tasks overlap but are not interchangeable. Two concordant deep cultures of the same organism provide strong microbiological support for PJI, whereas a single detection can have different meaning according to organism virulence, specimen type, prior antibiotics, and the remaining diagnostic evidence (Osmon et al., 2013; Parvizi et al., 2011, 2018; McNally et al., 2021; Atkins et al., 1998). Once infection is already established by a sinus tract, histology, synovial findings, or a dominant organism, a second low-confidence signal does not automatically become causal merely because the episode is unquestionably infected.

This distinction is especially important in biofilm disease. Organisms may be spatially segregated on the implant or present at unequal abundance (Stoodley et al., 2008; Peters et al., 2012; Stacy et al., 2016). General biofilm biology provides biological plausibility for heterogeneous recovery but cannot by itself establish causal participation of an additional organism. Conversely, a skin commensal recovered once during a complex revision remains susceptible to contamination. The appropriate unit of reasoning is therefore the organism within the episode, not the episode alone.

## 4 Why multiple detections do not necessarily indicate true polymicrobial infection

Mixed results can arise before, during, or after analytical testing. Superficial wounds and sinus tracts may carry organisms that differ from the deep implant community. Intraoperative contamination can occur through skin, instruments, containers, transport, or laboratory handling. Pooling specimens destroys the independence required to distinguish repeated recovery from a single contamination event. Prior antibiotics can selectively suppress susceptible or abundant organisms and reveal only part of the community, producing apparent discordance between preoperative and intraoperative cultures (Osmon et al., 2013; Berbari et al., 2007).

The temporal boundary of an infection episode also matters. Two organisms recovered during the same operation are not necessarily simultaneous biological partners, and organisms recovered weeks apart may represent persistence, ecological succession under antibiotic pressure, hematogenous reseeding, or a genuinely new infection. Open wound management and repeated procedures can permit sequential colonization and make a flat list of isolates particularly misleading (Valenzuela et al., 2022). Episode definitions should therefore record the date, procedure, anatomical source, and treatment exposure associated with every isolate.

Molecular methods create additional ambiguity. PCR and metagenomic sequencing may detect difficult-to-culture organisms or organisms rendered nonviable by treatment, but low-biomass samples are highly vulnerable to reagent and laboratory contamination (Street et al., 2017; Thoendel et al., 2018; Salter et al., 2014; Eisenhofer et al., 2019). Sequence abundance is not equivalent to viable burden, and database or reporting thresholds can turn background signal into a named organism. The solution is neither to dismiss molecular detections nor to accept every read as a pathogen; it is to interpret each result against sampling quality, controls, reproducibility, and clinical plausibility.

## 5 Evidence domains for organism-level causal attribution

**Figure 1:**
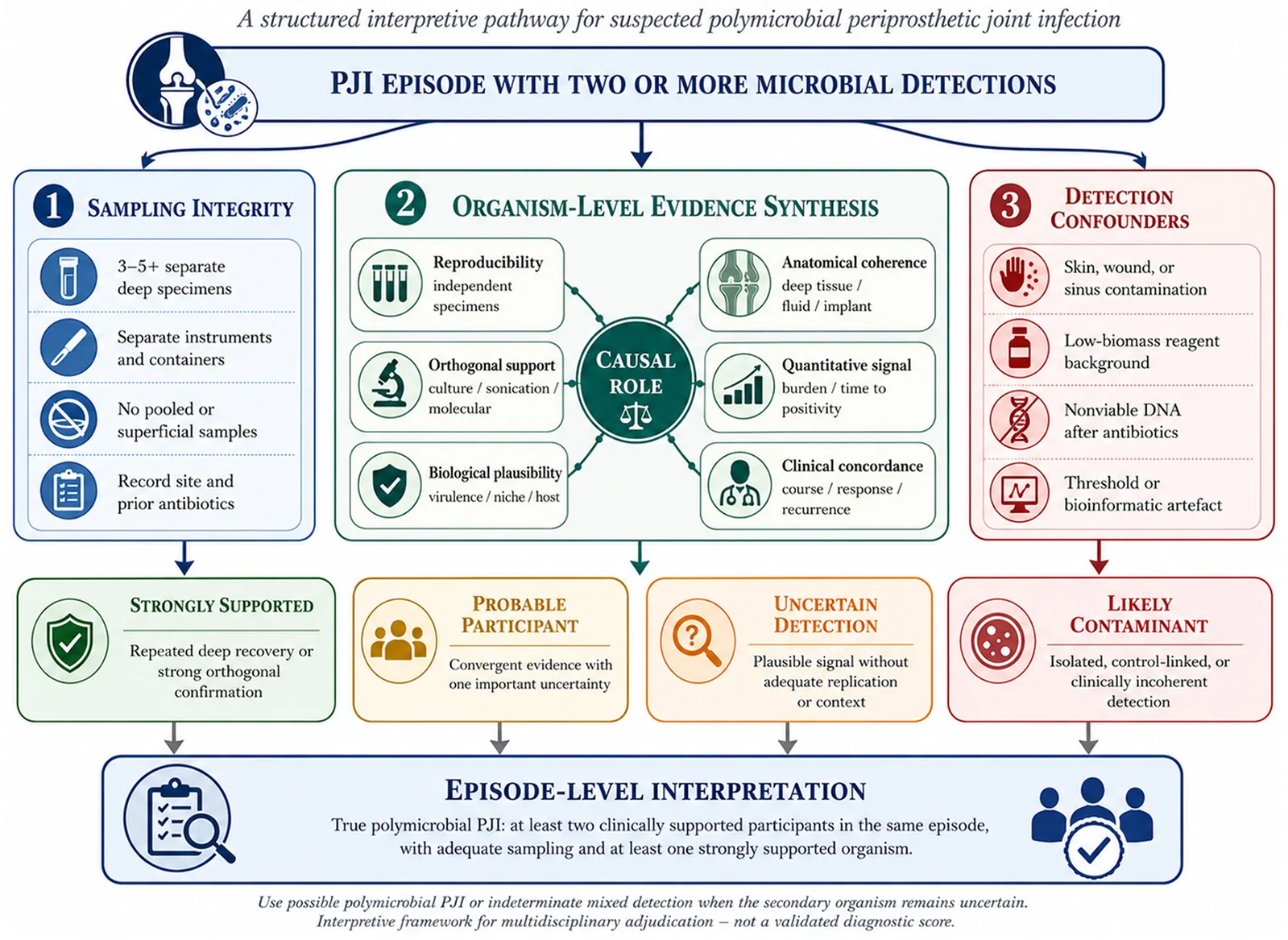
From multiple microbial detections to organism-level causality in PJI. Adequate independent deep sampling is a gatekeeping requirement. Each detected organism is then interpreted using reproducibility, anatomical coherence, orthogonal confirmation, quantitative and temporal signal, biological plausibility, and clinical concordance, while accounting for contamination, nonviable DNA, and analytical artifacts. The four organism-level categories and episode-level definition are proposed as transparent interpretive language, not as a validated diagnostic score.

The key evidence domains are summarized in Figure 1 and Table 1 and detailed below. Sampling integrity is the foundation. Multiple separate deep specimens obtained with separate instruments and placed in separate containers preserve evidentiary independence. Prospective and subsequent studies support obtaining several tissue specimens rather than relying on one or two samples (Atkins et al., 1998; Peel et al., 2017). When this standard is not met, uncertainty should be reported explicitly instead of being concealed by a confident polymicrobial label.

**Table 1:** Evidence domains that strengthen or weaken causal attribution of an organism in PJI.

Evidence domain	Findings supporting causal participation	Findings weakening causal participation	Important caveat
Sampling integrity	Several separate deep specimens; separate instruments and containers; adequate incubation.	One specimen, pooled material, superficial or sinus-tract sample, unclear handling.	Poor sampling creates uncertainty that sensitive downstream tests cannot fully correct.
Reproducibility	Same organism in independent deep specimens or repeated at separate time points.	Single isolated detection without replication.	True low-abundance or spatially restricted organisms may still be recovered once.
Anatomical coherence	Concordance among deep tissue, synovial fluid, implant sonication, or blood in a compatible acute episode.	Detection limited to a superficial route or a compartment inconsistent with the syndrome.	Spatial heterogeneity can produce real discordance across implant niches.
Orthogonal confirmation	Culture plus sonication, PCR, sequencing, histology, or organism-consistent bacteremia.	Only one analytical platform, especially without appropriate controls.	Shared contamination can create false concordance if workflows are not independent.
Quantitative and temporal signal	Multiple positive specimens, substantial burden, early positivity, persistence, or organism-specific recurrence.	Trace abundance, late isolated growth, or signal also present in controls.	No universal quantitative threshold applies across organisms and platforms.
Biological and clinical plausibility	Organism virulence, wound context, antibiotic exposure, host factors, and treatment course are coherent.	No plausible entry route, incompatible course, or disappearance without active coverage.	Taxonomic reputation alone cannot classify a commensal as contamination or a virulent isolate as causal.

Reproducibility is the strongest general signal. Recovery of the same organism from more than one independent deep specimen, or concordance between tissue culture and sonication, blood culture, PCR, or metagenomic sequencing, substantially increases causal credibility (Trampuz et al., 2007; Atkins et al., 1998; Peel et al., 2017; Street et al., 2017; Thoendel et al., 2018). However, concordance is not absolute proof: the same contaminant can recur through a shared laboratory source, and a genuine low-abundance organism may appear in only one compartment.

Anatomical coherence matters. Deep periprosthetic tissue, synovial fluid, sonication fluid, and blood during acute hematogenous infection provide different windows on the episode. A sinus-tract swab or superficial wound sample has weaker attribution value because it samples a colonized route rather than the implant interface. Discordance between compartments should be explained biologically before it is labeled contamination or a new infection.

Quantitative and temporal signals add context. The number of positive specimens, colony burden, time to positivity, molecular read count, persistence across time, and organism-specific recurrence can all strengthen or weaken attribution. None has a universal threshold across platforms. A high-virulence organism detected once may be more credible than a typical contaminant found at trace abundance, but taxonomic reputation must not replace episode-specific evidence. Coagulase-negative staphylococci, Cutibacterium species, coryneform bacteria, and other commensals can be authentic implant pathogens when repeatedly recovered from high-quality specimens (Zimmerli et al., 2004; Tande and Patel, 2014; Del Pozo and Patel, 2009; Izakovicova et al., 2019).

Finally, clinical concordance includes timing, wound condition, sinus tract, bacteremia, remote infection source, host vulnerability, antimicrobial exposure, and response. Enterococci and gram-negative bacilli are disproportionately represented in difficult and polymicrobial episodes, but their presence can also mark complex host and surgical conditions rather than independently determine failure (Tornero et al., 2014; Renz et al., 2019; Kheir et al., 2017; Hsieh et al., 2009; Rodríguez-Pardo et al., 2014). Organism-level causality is strongest when microbiological, anatomical, and clinical evidence converge.

Organism identity should modify, but not dictate, interpretation. A single deep detection of *Staphylococcus aureus*, a beta-hemolytic *Streptococcus*, an *Enterobacterales* species, or Pseudomonas aeruginosa may carry more prior probability than a single detection of a common skin commensal. Nevertheless, even a traditionally virulent organism can be introduced during sampling, and repeatedly recovered commensals can be genuine biofilm pathogens. The evidentiary standard should therefore be asymmetric rather than taxonomically absolute: virulence changes the amount of corroboration required, not whether corroboration is required at all.

## 6 Interpreting common diagnostic modalities

Tissue culture remains the principal method because it recovers viable organisms and enables susceptibility testing. Its value depends on specimen number and independence, incubation strategy, and prior antimicrobial exposure (Atkins et al., 1998; Peel et al., 2017). A single positive low-virulence isolate should not be interpreted without the wider episode, while repeated recovery from separate deep specimens is strong evidence.

Synovial fluid culture samples the joint space but may not reproduce the organisms embedded on the implant. It is most persuasive when concordant with deep tissue or blood culture and when obtained before antibiotics. Blood culture is particularly informative in acute hematogenous PJI, although a bloodstream organism must still be related anatomically and temporally to the joint.

Sonication dislodges organisms from removed components and can increase sensitivity, especially after antibiotics (Trampuz et al., 2007). Quantitative thresholds and container handling are important because sonication fluid can also acquire contamination. A sonication-only organism is more credible when its burden is substantial or when supported by tissue, molecular, or clinical evidence.

PCR and metagenomic sequencing can disclose suppressed or uncultivable organisms, but they detect nucleic acid rather than necessarily viable, disease-driving microbes (Street et al., 2017; Thoendel et al., 2018). Negative extraction controls, batch controls, platform-specific thresholds, and reporting of absolute or normalized abundance are essential in low-biomass orthopedic samples (Salter et al., 2014; Eisenhofer et al., 2019). Molecular methods should increase resolution, not eliminate clinical adjudication. The choice of specimen critically influences molecular yield and interpretability. Periprosthetic tissue provides direct access to the biofilm niche but may contain PCR inhibitors; synovial fluid offers a relatively clean matrix with fewer inhibitors and is the preferred specimen for targeted PCR when available; sonication fluid dislodges adherent organisms and can increase sensitivity, particularly for organisms embedded in the biofilm that may not be captured by fluid aspiration alone. Each specimen type carries distinct pre-analytical considerations: tissue requires mechanical homogenization, synovial fluid may be limited in volume in dry taps, and sonication fluid demands rigorous container handling to avoid exogenous DNA introduction. Prior antimicrobial exposure is a major confounder for all diagnostic methods, but it affects culture and molecular techniques differently. Antibiotics suppress viable growth and can render cultures falsely negative within hours to days of administration, while microbial DNA may persist in the joint space for days to weeks after organism death. This creates a characteristic discordance pattern—culture-negative, sequence-positive results—that is especially common when specimens are obtained after empiric antibiotic initiation. Conversely, prolonged suppressive therapy can reduce the organism biomass below the detection limit of both culture and sequencing, generating double-negative results despite ongoing infection. Molecular methods provide incremental value in several well-defined clinical scenarios: (i) culture-negative PJI where standard microbiological workup is unrevealing despite strong clinical and histological evidence of infection; (ii) prior antibiotic exposure that may have suppressed cultivable organisms; (iii) detection of fastidious or slow-growing organisms such as Cutibacterium species, *Peptostreptococcus*, or *Kingella* that require extended incubation or special culture conditions; (iv) discordance between preoperative aspiration and intraoperative tissue cultures where molecular testing can adjudicate whether the discrepant signals represent contamination, sampling of different niches, or treatment-driven selection; and (v) suspected polymicrobial infection where culture may selectively recover only the most abundant or fastest-growing members. Cross-method discordance should be interpreted explicitly. Culture-positive and sequence-negative results may reflect low nucleic-acid yield, incomplete databases, or stochastic detection, whereas sequence-positive and culture-negative results may reflect antimicrobial suppression, fastidious growth, nonviable DNA, or contamination. A positive molecular signal without culture confirmation should not automatically broaden antimicrobial therapy. Interpretation should incorporate extraction and batch controls, signal abundance, prior antimicrobial exposure, and corroborating evidence from targeted PCR, repeat deep sampling, histology, or compatible clinical findings. When a molecular-only organism would materially broaden antimicrobial coverage, prolong treatment, or alter surgical strategy, confirmatory testing and multidisciplinary review involving infectious diseases, orthopedic surgery, and clinical microbiology are appropriate before the detection is accepted as causally relevant.

## 7 A pragmatic vocabulary for mixed detections

We propose a four-category vocabulary to make organism-level causal confidence explicit in multidisciplinary interpretation and research reporting (Figure 1, Table 1). It is an interpretive framework rather than a validated diagnostic score. A strongly supported participant is repeatedly recovered from independent deep specimens or has strong orthogonal confirmation and clinical coherence. A probable participant has convergent evidence but retains one important uncertainty. An uncertain detection is biologically plausible yet insufficiently reproducible or contextualized. A likely contaminant is an isolated, low-confidence, or control-linked signal without anatomical or clinical coherence.

At the episode level, the term true polymicrobial PJI should be reserved for infection in which at least two organisms are clinically supported participants in the same episode, sampling is adequate, and at least one organism is strongly supported. When the second organism remains uncertain, terms such as possible polymicrobial PJI or indeterminate mixed detection are more transparent. The vocabulary complements rather than replaces validated PJI definitions.

Several recurring scenarios illustrate the value of graded language. Four of five tissues growing *S. aureus* with one isolated coagulase-negative staphylococcus usually supports a dominant pathogen and an uncertain secondary detection unless the latter is independently confirmed. Three different low-virulence organisms from three specimens may indicate poor sampling or contamination rather than a three-member community. A culture-confirmed staphylococcal PJI with a low-abundance gram-negative mNGS signal requires scrutiny of controls, abundance, prior antibiotics, and targeted confirmation. Discordant preoperative aspiration and intraoperative tissue cultures after antibiotic exposure may represent sampling of different niches or treatment-driven selection rather than laboratory error. These examples cannot be solved by organism counting alone.

## 8 Discussion

The proposed vocabulary organizes commonly used evidentiary considerations into explicit organism-level categories that can be documented and evaluated prospectively. Its immediate contribution is interpretive transparency and reproducibility of reasoning, not a demonstrated improvement in antimicrobial use or patient outcomes.

Overcalling polymicrobial infection can trigger unnecessary gram-negative, enterococcal, anaerobic, or antifungal coverage; increase renal, hepatic, hematological, and microbiome toxicity; complicate oral step-down therapy; and promote resistance. This is particularly consequential when a single commensal or molecular signal is added to a well-supported dominant pathogen. Broader therapy is not intrinsically more precise. Undercalling is also hazardous. A credible secondary organism left untreated may cause breakthrough infection, persistence after debridement, or recurrence after exchange. Studies of polymicrobial, enterococcal, and gram-negative PJI demonstrate the treatment complexity and poorer outcomes that can occur when difficult organisms participate (Marculescu and Cantey, 2008; Tan et al., 2016; Li et al., 2021; Flurin et al., 2019; Wimmer et al., 2016; Bozhkova et al., 2016; Auñón et al., 2025; Chahla et al., 2019; Valenzuela et al., 2022; Tornero et al., 2014; Renz et al., 2019; Kheir et al., 2017; Hsieh et al., 2009; Rodríguez-Pardo et al., 2014; Li et al., 2022). The goal is calibrated coverage: treat organisms with credible causal evidence, seek confirmation when uncertainty is consequential, and align antimicrobial decisions with source control rather than with a flat list of laboratory detections.

Causal attribution also affects surgery. A disputed second organism may influence whether a one-stage exchange is considered microbiologically predictable, whether local antibiotic formulations are broadened, and how reimplantation cultures are interpreted. Conversely, labeling every additional isolate as a pathogen can make apparently successful source control look like microbiological failure. Multidisciplinary decisions should document which organisms are being treated as causal, which are being monitored, and what new evidence would change that judgment.

## 9 Future perspectives and reporting standards

Looking forward, several research priorities emerge. Future studies should preserve specimen-level microbiology. At minimum, authors should report the number and anatomical source of specimens, instruments and containers used, antibiotic exposure before sampling, organism-specific positive specimen counts, time to positivity or colony burden, sonication thresholds, molecular controls, abundance metrics, susceptibility results, and organism-specific treatment coverage. Sequential isolates should be classified as persistence, recurrence, newly revealed community member, or new infection when evidence permits. Prospective multicenter validation should compare organism-level classifications with blinded multidisciplinary adjudication, breakthrough infection, organism-specific recurrence, treatment failure, and inter-rater reliability. Importantly, these studies must avoid circularity: an organism should not be declared causal solely because therapy was directed against it, nor dismissed because broad therapy happened to suppress it. Consensus treatment-success definitions can standardize outcome assessment (Diaz-Ledezma et al., 2013). A useful validation design would freeze the organism-level classification before definitive therapy, retain isolates and residual specimens, and repeat adjudication after follow-up without allowing outcome knowledge to alter the baseline classification. Calibration should be tested separately for culture-only, culture-plus-sonication, and sequencing-enriched workflows. Prospective studies should test whether the framework improves reproducibility, predicts organism-specific failure, or reduces unnecessary antimicrobial exposure beyond existing episode-level PJI definitions. These outcomes are validation hypotheses rather than established benefits.

True polymicrobial PJI is not defined adequately by the arithmetic of culture reports. It is an episode-level ecological diagnosis built from organism-level evidence. Sampling quality acts as a gatekeeper; reproducibility, anatomical coherence, orthogonal confirmation, quantitative signal, biological plausibility, and clinical course then determine how strongly each organism can be linked to infection. The principal controversy is how much evidence is enough. A very strict requirement for repeated culture may miss suppressed or spatially restricted participants, whereas permissive acceptance of every molecular signal will inflate false polymicrobial diagnoses. No universal rule resolves this tension. Transparent graded language is therefore preferable to an unsupported binary label until prospective validation is available. The practical objective is not to create another unvalidated score, but to make uncertainty visible and clinically actionable.

## 10 Conclusion

A true polymicrobial PJI should comprise at least two organisms with credible causal participation in the same infected joint episode, not merely two names on a report. High-quality independent sampling, repeated or orthogonal detection, anatomical and quantitative coherence, organism biology, and clinical context should be integrated for each microorganism. This framework provides transparent language for organism-level attribution and a reproducible foundation for prospective validation. Whether it improves antimicrobial precision or prevents organism-specific treatment failure remains to be tested.
